# Change in skeletal muscle mass is associated with hepatic steatosis in nonalcoholic fatty liver disease

**DOI:** 10.1038/s41598-023-34263-z

**Published:** 2023-04-28

**Authors:** Ik Hyun Jo, Do Seon Song, U Im Chang, Jin Mo Yang

**Affiliations:** Department of Internal Medicine, St. Vincent’s Hospital, College of Medicine, The Catholic University of Korea, 93 Jungbu-Daero, Paldal-Gu, Suwon, Gyeonggi-Do 16247 Republic of Korea

**Keywords:** Hepatology, Non-alcoholic fatty liver disease

## Abstract

The association between nonalcoholic fatty liver disease (NAFLD) and sarcopenia is known. We aimed to determine the association between skeletal muscle mass changes and NAFLD status. This retrospective single-center study analyzed patients who underwent health screening twice between November 2009 and December 2017, with a temporal gap of 6 ± 0.5 years. The degree of sarcopenia was assessed using appendicular skeletal muscle mass (ASM) adjusted for weight and body mass index (BMI). Changes in hepatic steatosis and fibrosis status were evaluated using noninvasive serum markers. Patients with a decrease in ASM/BMI (n = 353) had increased hepatic steatosis index (HSI) and fatty liver index (FLI) scores during 6 years (p < 0.05). The baseline sarcopenia group had a greater elevation in NAFLD fibrosis score (NFS) over 6 years than those without baseline sarcopenia. ASM changes over 6 years showed a negative correlation with variations in HSI (β = − 0.96 in ASM/Weight and -28.93 in ASM/BMI) and FLI (β = − 5.44 in ASM/Weight and − 167.12 in ASM/BMI). Subgroup analyses showed similar results according to sex and age. Sarcopenia may worsen steatosis and vice versa. Skeletal muscle status can be used to predict the course of NAFLD and establish individualized treatment strategies.

## Introduction

Nonalcoholic fatty liver disease (NAFLD), characterized by hepatic fat accumulation in the absence of excessive alcohol consumption, is the most common cause of chronic liver disease worldwide^1,2^. NAFLD is a spectrum including isolated hepatic steatosis, nonalcoholic steatohepatitis (NASH), fibrosis, and ultimately cirrhosis^3^. NAFLD is associated with increased liver-related mortality, including complications of portal hypertension, liver failure, and hepatocellular carcinoma^4–6^. Furthermore, NAFLD is associated with cardiovascular disease and malignancy-related mortality^7^. Additionally, the most important histologic feature—which determines long-term prognosis—is fibrosis stage rather than hepatic inflammation^8,9^. Therefore, improving hepatic fibrosis is considered an important surrogate endpoint of NAFLD patients^10^.

A previous study has shown that NAFLD is closely associated with insulin resistance^11^. Since skeletal muscle is the major site for glucose disposal by stimulation of insulin, low skeletal muscle mass promotes insulin resistance independent of obesity, leading to hepatic steatosis^12,13^. Moreover, NAFLD is a chronic low grade inflammatory state with increased circulating inflammatory cytokines, which might cause muscle depletion^12^. Recent studies have shown that, in patients with NAFLD, sarcopenia is significantly associated with steatohepatitis and fibrosis, independent of obesity and insulin resistance^14^. However, because these were cross-sectional studies, it is difficult to establish the causal relationship between sarcopenia and NAFLD. Although a large cohort study showed that skeletal muscle mass change was associated with the development of NAFLD or resolution of pre-existing NAFLD^15^, there is a lack of studies showing the association between changes in muscle mass and intrahepatic fibrosis.

Therefore, we aimed to investigate whether changes in muscle mass affect changes in hepatic steatosis and fibrosis, measured by noninvasive serum markers in NAFLD patients.

## Methods

### Study population

This was a retrospective study conducted in St. Vincent`s Hospital, the Catholic University of Korea, which reviewed the medical records of health screening between November 2009 and December 2017. Those who had received health screening more than twice in our center, who were diagnosed with fatty liver through abdominal ultrasound (US) were included. The ACUSON Sequoia 512 (Siemens Medical Solution, Mountain View, CA, USA) or EPIQ 5 US system (Philips Ultrasound, Bothell, WA, USA) were used for abdominal US, and the examination was performed by experienced radiologists.

There were 59,886 health screening cases during the study period. Patients without fatty liver on US were excluded (n = 39,667). Additionally, cases with hepatitis B surface antigen or undetermined hepatitis B surface antigen (n = 894), hepatitis C antibody (n = 84), liver cirrhosis (n = 99), insufficient medical records (n = 7755), a history of concomitant malignancy (n = 338), and excessive alcohol consumption (≥ 30 g/day for men, ≥ 20 g/day for women, n = 1078) were excluded.

**Figure 1:**
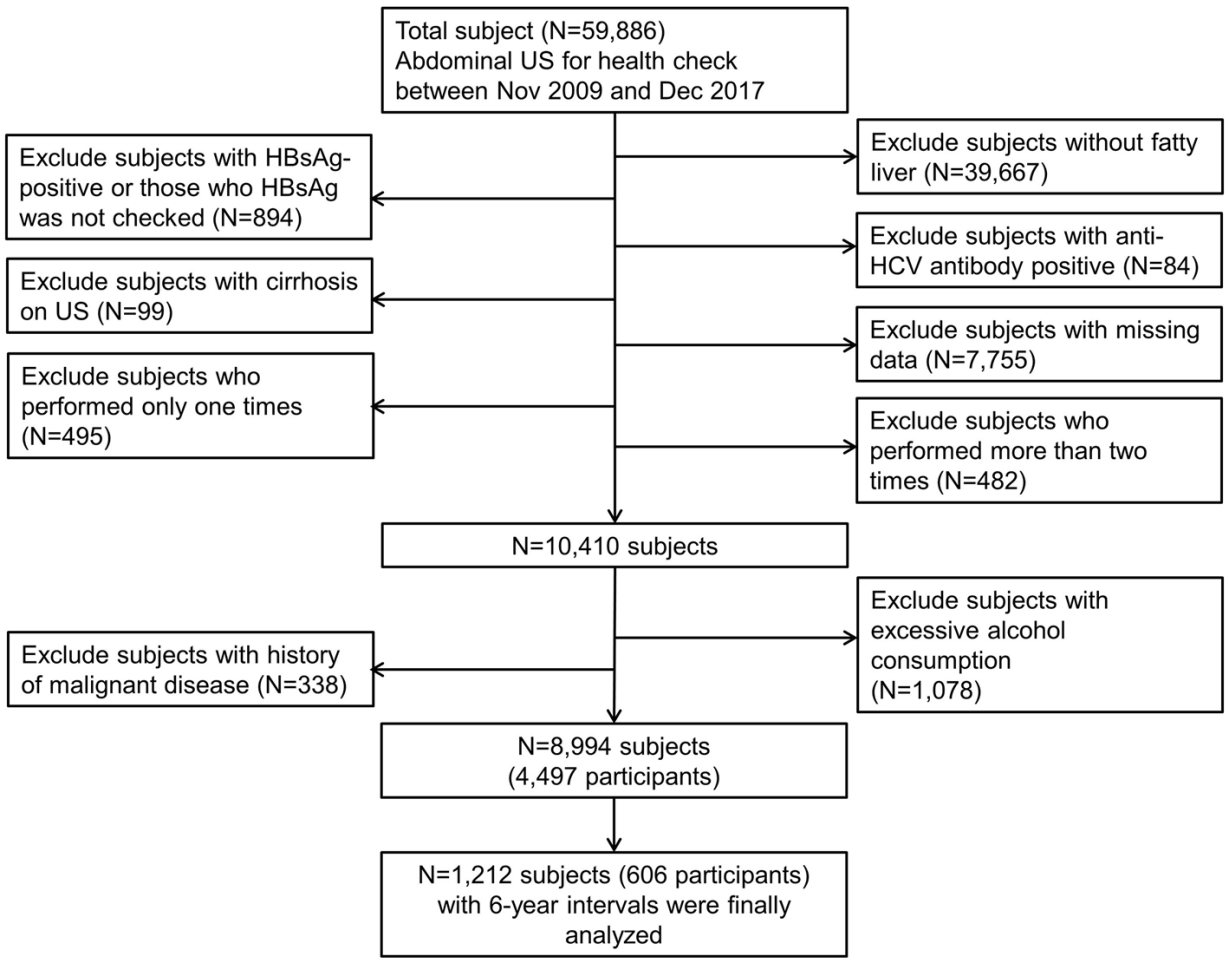
Flowchart for the composition of the study participants.

Finally, the data of 606 patients were analyzed by limiting the subjects to those who received health screening only twice at an interval of 5.5–6.5 years. The enrollment process for the study population is shown in Fig. 1. The participants’ demographics, height, weight, comorbid diseases, social history, and results of various blood tests performed during screening were investigated. This study complies with the Declaration of Helsinki. Due to the retrospective design, we did not obtain informed consent for each participant. Ethical approval including the informed consent waiver, and monitoring of details of the research process was performed by the institutional review board of St. Vincent`s Hospital (VC17RESI0213).

### Muscle mass parameters and definition of sarcopenia

The appendicular skeletal muscle mass (ASM) was obtained by adding the skeletal muscle mass of each limb measured by simultaneous multifrequency impedance measurements (Inbody770, Inbody Co., Korea). Sarcopenia was evaluated using the following two indices normalizing ASM with body weight and body mass index (BMI): ASM/weight and ASM/BMI. The definitions of sarcopenia were as follows: (1) ASM/weight < 2 standard deviation below the mean in healthy young adults, according to nationwide health examinations of the Korean population (sex-specific, < 29.0% in males or < 22.9% in females)^16^, (2) ASM/BMI < 0.789 m^2^ in males, or < 0.512 m^2^ in females, respectively^17^.

### Concomitant metabolic disorders

Hypertension was defined as systolic blood pressure ≥ 140 mmHg, diastolic blood pressure ≥ 90 mmHg, or a history of antihypertensive treatment. Those with a fasting serum glucose level ≥ 126 mg/dL or a history of hypoglycemic agent use were defined as having diabetes mellitus^18^. Metabolic syndrome was diagnosed when three or more of the following requirements were met: (1) waist circumference: male, > 90 cm; female, > 80 cm; (2) triglyceride ≥ 150 mg/dL or ongoing drug treatment for hypertriglyceridemia; (3) high-density lipoprotein (HDL) cholesterol: male, < 40 mg/dL; female, < 50 mg/dL or ongoing drug treatment for reduced HDL cholesterol; (4) systolic blood pressure ≥ 130 mmHg or diastolic blood pressure ≥ 85 mmHg or ongoing antihypertensive medications; (5) fasting serum glucose ≥ 100 mg/dL or ongoing drug treatment for elevated blood glucose^19,20^.

### Measuring hepatic steatosis and fibrosis

Based on the screening results, the following four noninvasive serum markers at two separate visits were calculated: NAFLD fibrosis score (NFS), Fibrosis-4 index for liver fibrosis (FIB4), hepatic steatosis index (HSI), and fatty liver index (FLI)^21–24^. Among them, NFS and FIB4 are scales used to predict the presence or extent of hepatic fibrosis, whereas HSI and FLI are scales evaluating the degree of hepatic steatosis. At the second visit (after 6 years), we calculated the variance (delta, Δ) of the four indices from the first visit and attempted to find correlations with sarcopenia.

### Statistical analysis

Data are expressed as means with standard deviations or numbers with percentages for continuous and categorical variables, respectively. Differences between the two groups were analyzed using t-tests and chi-square tests. Regression analyses were performed for correlations between muscle mass parameters and liver-related indices (adjusting for age, sex, concomitant diabetes, hypertension, and metabolic syndrome were adjusted). All data analyses were performed using the R Studio software for data analysis (ver. 1.0.153; R Foundation for Statistical Computing, Vienna, Austria). p values < 0.05 were considered statistically significant.

## Results

### Baseline characteristics of the study population

Data from 606 subjects were analyzed. The median age of the subjects at the first visit was 47 years, and the male sex was dominant (67.8%). Among the study population, 67 subjects (11.1%) had diabetes, 204 (33.7%) had hypertension, and 247 (40.8%) had metabolic syndrome at the first visit. Sixty-three participants (10.4%) met the criteria for sarcopenia based on ASM/weight or ASM/BMI. The group with sarcopenia (meeting criteria according to either ASM/weight or ASM/BMI) at the first visit showed a significantly higher BMI (p < 0.05) and higher proportion of hypertension and metabolic syndrome than the others. Laboratory findings showed that white blood cells (WBC) count, hemoglobin, aspartate aminotransferase (AST), alanine aminotransferase (ALT), and triglycerides were significantly higher (p < 0.05) in the sarcopenia group than in the non-sarcopenic group. The baseline characteristics of the study population and details of the differences according to the presence of sarcopenia are described in Table 1.

**Table 1:** Baseline characteristics by initial sarcopenia status (bold: p value < 0.05).

	Total (n = 606)	Normal (n = 543)	Sarcopenia* (n = 63)	p value
Age (years)	47.3 ± 9.1	47.1 ± 8.8	49.4 ± 11.2	0.116
Gender: male	411 (67.8%)	363 (66.9%)	48 (76.2%)	0.174
Interval (years)	6.0 ± 0.2	6.0 ± 0.2	6.0 ± 0.2	0.853
Body mass index (kg/m^2^)	25.3 ± 2.8	**24.9 ± 2.6**	**28.2 ± 3.0**	** < 0.001**
Diabetes	67 (11.1%)	60 (11.0%)	7 (11.1%)	1.000
Hypertension	204 (33.7%)	**174 (32.0%)**	**30 (47.6%)**	**0.020**
Metabolic syndrome	247 (40.8%)	**208 (38.3%)**	**39 (61.9%)**	**0.001**
Smoking: Yes	151 (24.9%)	132 (24.3%)	19 (30.2%)	0.389
Alcohol use: Yes	326 (53.8%)	295 (54.3%)	31 (49.2%)	0.523
Laboratory findings				
WBC (10^9^/L)	6.2 ± 1.7	**6.1 ± 1.6**	**6.9 ± 1.9**	** < 0.001**
Hemoglobin (g/dL)	14.7 ± 1.6	**14.6 ± 1.6**	**15.1 ± 1.4**	**0.025**
Platelet (10^9^/L)	253.9 ± 56.6	252.9 ± 56.0	262.5 ± 61.3	0.202
Glucose (mg/dL)	99.6 ± 25.6	99.7 ± 26.5	98.3 ± 15.1	0.534
Protein (g/dL)	7.3 ± 0.4	7.3 ± 0.4	7.3 ± 0.4	0.906
Albumin (g/dL)	4.6 ± 0.3	4.6 ± 0.3	4.5 ± 0.3	0.246
BUN (mg/dL)	14.8 ± 3.9	14.8 ± 3.8	15.0 ± 4.3	0.709
Creatinine (mg/dL)	0.9 ± 0.5	0.9 ± 0.5	0.9 ± 0.2	0.496
Sodium (mEq/L)	142.1 ± 1.9	142.1 ± 1.9	142.1 ± 1.8	0.918
Potassium (mEq/L)	4.3 ± 0.3	4.3 ± 0.3	4.3 ± 0.3	0.910
Total bilirubin (mg/dL)	0.9 ± 0.3	**0.9 ± 0.4**	**0.8 ± 0.3**	**0.024**
AST (IU/L)	22.5 ± 8.9	**22.2 ± 8.9**	**25.2 ± 8.7**	**0.010**
ALT (IU/L)	27.7 ± 17.6	**26.5 ± 16.5**	**37.9 ± 23.4**	** < 0.001**
ALP (IU/L)	195.4 ± 50.6	194.7 ± 50.5	201.9 ± 51.3	0.287
GGT (U/L)	35.3 ± 38.7	34.5 ± 39.6	42.4 ± 29.2	0.056
Uric acid (mg/dL)	5.6 ± 1.5	5.6 ± 1.4	5.8 ± 1.7	0.279
Total cholesterol (mg/dL)	206.3 ± 38.3	205.6 ± 37.9	212.7 ± 40.7	0.161
Triglyceride (mg/dL)	145.3 ± 101.1	**140.3 ± 92.0**	**187.8 ± 154.1**	**0.019**
HDL cholesterol (mg/dL)	45.2 ± 10.0	45.4 ± 10.1	43.1 ± 8.5	0.075
LDL cholesterol (mg/dL)	126.8 ± 33.3	126.3 ± 33.3	131.0 ± 33.8	0.294

*WBC* White blood cell, *BUN* Blood urea nitrogen, *AST* Aspartate aminotransferase, *ALT* Alanine aminotransferase, *ALP* Alkaline phosphatase, *GGT* Gamma-glutamyl transferase, *HDL* High-density lipoprotein, *LDL* Low-density lipoprotein.

*The presence of sarcopenia was determined by criteria based on ASM/Weight and ASM/BMI.

### State of sarcopenia and NAFLD indices at the baseline and 6 years later

The ASM/weight and ASM/BMI of the study population did not change significantly over 6 years. However, the proportion of sarcopenia showed a 6-year increasing trend from the baseline. Among the liver-related indices, NFS and FIB4 decreased significantly over 6 years (p < 0.05). Furthermore, HSI increased after 6 years, while FLI showed a statistically insignificant decreasing pattern (Table 2).

**Table 2:** Changes in muscle mass and sarcopenia status for 6 years (bold: p value < 0.05).

	Baseline (t_0_)	After 6 years (t_1_)	p value
ASM (kg)	21.1 ± 4.4	21.1 ± 4.4	0.989
ASM/weight (%)	29.7 ± 3.5	30.0 ± 3.5	0.124
ASM/BMI (m^2^)	0.8 ± 0.2	0.8 ± 0.2	0.233
Sarcopenia: Yes	63 (10.4%)	78 (12.9%)	0.210
Sarcopenia			0.494
By ASM/weight	21 (3.5%)	22 (3.6%)	
By ASM/BMI	9 (1.5%)	14 (2.3%)	
Both	33 (5.4%)	42 (6.9%)	
Liver-related index			
NFS	**− 2.1 ± 1.2**	**− 2.5 ± 1.2**	**< 0.001**
FIB4	**1.2 ± 0.5**	**0.9 ± 0.4**	**< 0.001**
HSI	**34.8 ± 4.6**	**35.6 ± 4.9**	**0.003**
FLI	38.1 ± 23.1	37.0 ± 24.2	0.461

*ASM* Appendicular skeletal muscle mass, *BMI* Body mass index, *NFS* NAFLD Fibrosis Score, *FIB4* Fibrosis-4 Index for Liver Fibrosis, *HIS* Hepatic Steatosis Index, *FLI* Fatty Liver Index.

Based on the changes in ASM/BMI, we divided all patients into two groups: those who showed a decrease in muscle mass over 6 years and those who did not. Approximately 58% of the subjects showed a decrease in the ASM/BMI over 6 years. When comparing clinical factors at the time of the first visit, the prevalence of metabolic syndrome was lower in the group with decreased ASM/BMI than the group with no decrease in ASM/BMI. Additionally, over the 6 years, the HSI and FLI showed a significant increase in the group with decreased ASM/BMI (p < 0.05). Regarding the laboratory findings, WBC count, hemoglobin, fasting glucose, total protein, albumin, AST, ALT, and γ-glutamyl transferase (GGT) levels showed a greater increase in the group with decreased ASM/BMI. The total cholesterol and triglyceride levels decreased in both groups; however, the variation was smaller in the group with decreased ASM/BMI. Furthermore, low-density lipoprotein (LDL) cholesterol levels increased over 6 years in the group with decreased ASM/BMI (Table 3). We performed the same analysis for the subgroup of 42 patients with sarcopenia by ASM/BMI during their baseline visit. Similar tendencies were seen throughout the population, but the statistical significance of the two groups could not be confirmed (Supplement Table S1).

**Table 3:** Differences of two groups according to the muscle mass reduction, at the time of baseline visit and changes for 6 years (bold: p value < 0.05).

	Reduced ASM/BMI (−) N = 253	Reduced ASM/BMI (+) N = 353	p
Age (years)	46.8 ± 8.8	47.7 ± 9.3	0.277
Gender: Male	77 (30.4%)	118 (33.4%)	0.490
BMI (kg/m^2^)	25.5 ± 2.6	25.1 ± 2.9	0.150
Diabetes	30 (11.9%)	37 (10.5%)	0.688
Hypertension	81 (32.0%)	123 (34.8%)	0.523
Metabolic syndrome	117 (46.2%)	130 (36.8%)	0.025
Smoking: Yes	58 (22.9%)	93 (26.3%)	0.387
Alcohol use: Yes	137 (54.2%)	189 (53.5%)	0.948
Changes for 6 years in liver-related index			
** Δ**NFS	0.5 ± 0.8	0.5 ± 0.8	0.430
** Δ**FIB4	0.3 ± 0.3	0.3 ± 0.3	0.107
** Δ**HSI	− 2.3 ± 3.2	0.3 ± 3.4	< 0.001
** Δ**FLI	− 6.3 ± 17.3	6.6 ± 15.5	< 0.001
Changes for 6 years in Laboratory findings			
ΔWBC (10^9^/L)	− 0.2 ± 1.4	0.3 ± 1.5	< 0.001
ΔHemoglobin (g/dL)	0.0 ± 1.1	0.4 ± 1.0	< 0.001
** Δ**Platelet (10^9^/L)	− 0.5 ± 38.9	5.3 ± 36.9	0.065
ΔGlucose (mg/dL)	1.4 ± 21.1	8.1 ± 22.2	< 0.001
ΔProtein (g/dL)	− 0.1 ± 0.4	0.0 ± 0.4	< 0.001
ΔAlbumin (g/dL)	− 0.1 ± 0.3	0.0 ± 0.3	0.006
** Δ**BUN (mg/dL)	− 1.3 ± 3.9	− 1.1 ± 4.4	0.512
** Δ**Creatinine (mg/dL)	− 0.1 ± 0.2	− 0.1 ± 0.3	0.564
** Δ**Sodium (mEq/L)	− 4.3 ± 19.9	− 2.8 ± 27.2	0.456
** Δ**Potassium (mEq/L)	− 0.2 ± 0.7	− 0.1 ± 0.9	0.365
** Δ**Total bilirubin (mg/dL)	0.1 ± 0.3	0.1 ± 0.3	0.472
ΔAST (IU/L)	3.4 ± 10.8	6.4 ± 11.4	0.001
ΔALT (IU/L)	− 3.3 ± 15.5	4.8 ± 17.8	< 0.001
** Δ**ALP (IU/L)	− 122.1 ± 62.5	− 127.6 ± 43.5	0.224
ΔGGT (U/L)	− 3.5 ± 24.4	2.4 ± 26.8	0.006
** Δ**Uric acid (mg/dL)	0.0 ± 1.0	0.0 ± 0.9	0.694
ΔTotal cholesterol (mg/dL)	− 19.1 ± 41.8	− 4.6 ± 41.5	< 0.001
ΔTriglyceride (mg/dL)	− 34.2 ± 97.7	− 3.0 ± 75.1	< 0.001
** Δ**HDL cholesterol (mg/dL)	6.2 ± 8.3	5.6 ± 8.0	0.356
ΔLDL cholesterol (mg/dL)	− 4.0 ± 35.6	7.0 ± 36.5	< 0.001

*ASM* Appendicular skeletal muscle mass, *BMI* Body mass index, *NFS* NAFLD Fibrosis Score, *FIB4* Fibrosis-4 Index for Liver Fibrosis, *HIS* Hepatic Steatosis Index, *FLI* Fatty Liver Index, *WBC* White blood cell, *BUN* Blood urea nitrogen, *AST* Aspartate aminotransferase, *ALT* Alanine aminotransferase, *ALP* Alkaline phosphatase, *GGT* Gamma-glutamyl transferase, *HDL* High-density lipoprotein, *LDL* Low-density lipoprotein.

### Changes in NAFLD indices according to sarcopenia status at baseline

Details regarding the changes in the four liver-related indices, according to the baseline sarcopenia status, are provided in Table 4. Based on both ASM/weight and ASM/BMI, the progression of NFS was greater in the sarcopenia group than in the non-sarcopenia group, indicating that the degree of hepatic fibrosis progression may become more severe in patients with sarcopenia than in those without. There were no significant differences in the changes in FIB4, HSI, and FLI. In addition, we conducted subgroup analyses according to sex and age (age > 50 years or < 50 years). The ΔNFS was significant in the male group (p < 0.05); however, no differences were observed among the four indices in the female group (Supplementary Table S2). In the subgroup analysis of the age groups, results similar to the entire study population were confirmed (Supplement Table S3).

**Table 4:** Changes in liver-related indices for 6 years according to the baseline sarcopenia status (Bold: p value < 0.05).

	ASM/weight			ASM/BMI		
	Sarcopenia (+)	Sarcopenia (−)	p	Sarcopenia (+)	Sarcopenia (−)	p
**Δ**NFS	0.8 ± 0.7	0.5 ± 0.8	0.002	0.8 ± 0.8	0.5 ± 0.8	0.003
**Δ**FIB4	0.3 ± 0.2	0.3 ± 0.3	0.945	0.3 ± 0.3	0.3 ± 0.3	0.773
**Δ**HSI	− 1.3 ± 3.6	− 0.8 ± 3.5	0.330	− 1.4 ± 3.8	− 0.8 ± 3.5	0.263
**Δ**FLI	− 2.1 ± 22.8	1.6 ± 16.8	0.246	− 1.3 ± 22.3	1.4 ± 17.0	0.441

*ASM* Appendicular skeletal muscle mass, *BMI* Body mass index, *NFS* NAFLD Fibrosis Score, *FIB4* Fibrosis-4 Index for Liver Fibrosis, *HIS* Hepatic Steatosis Index, *FLI* Fatty Liver Index.

### Prediction of the progression of steatosis and fibrosis using the change in muscle mass

We performed regression analyses to find out the relationship between changes in skeletal muscle mass and NAFLD indices. The increase in muscle mass showed a negative correlation with the changes in FLI and HSI over 6 years (Table 5). As muscle mass increased over the 6-year period, the degree of hepatic steatosis—reflected in FLI and HSI—also showed significant improvement (p < 0.05). In contrast, the hepatic fibrosis indicators NFS and FIB4 did not show any significant correlations with changes in muscle mass over 6 years.

**Table 5:** Regression analysis of muscle mass variances as predictors for changes in NAFLD-related indices over 6 years (bold: p value < 0.05).

	**Δ**ASM/weight				**Δ**ASM/BMI			
	Estimate	95% CI		p	Estimate	95% CI		p
		Lower limit	Upper limit			Lower limit	Upper limit	
Model 1								
** Δ**NFS	0.008	− 0.036	0.052	0.707	0.150	− 1.298	1.597	0.839
** Δ**FIB4	**0.018**	**0.002**	**0.035**	**0.033**	**0.613**	**0.283**	**2.167**	**0.031**
** Δ**HSI	**− 0.955**	**− 1.137**	**− 0.772**	**< 0.001**	**− 28.930**	**− 34.997**	**− 22.863**	**< 0.001**
** Δ**FLI	**− 5.441**	**− 6.305**	**− 4.578**	**< 0.001**	**− 167.116**	**− 195.932**	**− 138.300**	**< 0.001**
Model 2								
** Δ**NFS	0.018	− 0.026	0.062	0.419	0.469	− 0.984	1.920	0.527
** Δ**FIB4	0.015	− 0.002	0.032	0.076	0.512	− 0.048	1.072	0.073
** Δ**HSI	**− 0.920**	**− 1.103**	**− 0.737**	**< 0.001**	**− 27.964**	**− 34.045**	**− 21.882**	**< 0.001**
** Δ**FLI	**− 5.239**	**− 6.097**	**− 4.387**	**< 0.001**	**− 162.107**	**− 190.497**	**− 133.717**	**< 0.001**

*CI* Confidential interval, *ASM* Appendicular skeletal muscle mass, *BMI* Body mass index, *NFS* NAFLD Fibrosis Score, *FIB4* Fibrosis-4 Index for Liver Fibrosis, *HIS* Hepatic Steatosis Index, *FLI* Fatty Liver Index.

Model 1, adjusted by age and gender; Model 2, adjusted by age, gender, diabetes, hypertension, and metabolic syndrome.

We performed a subgroup regression analysis according to sex, age, and the presence of diabetes (Supplement Tables S4, S5 and S6, respectively). Regardless of sex, age, or concomitant diabetes, in the subgroup analysis, HSI and FLI showed a negative correlation with increasing ASM/weight and ASM/BMI, similar to the total study population. Furthermore, in this subgroup analysis, NFS and FIB4—which represent hepatic fibrosis—showed the same tendencies as the study population.

## Discussion

In this study, we investigated the relationship between the extent of skeletal muscle mass changes and hepatic fibrosis or steatosis in NAFLD patients. We found that patients with NAFLD and sarcopenia at the first visit had a higher BMI, as well as a higher prevalence of hypertension or metabolic syndrome. Additionally, patients with increased HSI and FLI showed decreased skeletal muscle mass over the 6-year period, as well as worsened serum glucose, lipid profile, and liver function. Whereas those with increased muscle mass over the 6-year period showed an improvement in hepatic steatosis, which was reflected in the FLI and HSI variances.

Our study used data from repetitive measurements in the same NAFLD patient group over a 6-year period; therefore, we were able to investigate quantitative changes in skeletal muscle mass, sarcopenia status, hepatic fibrosis, and steatosis over time. Meanwhile, abdominal US used for the diagnosis of NAFLD was performed by experienced experts in the referral hospital, which guaranteed the high quality of examination. Another strength of this study is that, in the process of evaluating hepatic steatosis or fibrosis, two different indices were used in an effort to reduce measurement method bias.

Our study results show that the exacerbation of sarcopenia and NAFLD are closely associated. Moreover, NAFLD and sarcopenia share many pathophysiological mechanisms, such as insulin resistance, chronic inflammatory conditions, and cell damage caused by oxidative stimulation^25^. Several previous studies have focused on the association between sarcopenia and chronic liver disease, especially NAFLD^26–28^. Of note in our study, the reduction in muscle mass exacerbated hepatic steatosis. Patients with sarcopenia at baseline had a higher rate of hepatic fibrosis progression, which was reflected in the change in NFS over 6 years. Similar results have been found in other studies^14–16^. A Korean longitudinal study similar to ours, with a 7-year observation period, indicated that an increase in skeletal muscle mass had a positive effect in slowing down the progression of NAFLD^15^. There are several explanations regarding how skeletal muscle gain is involved in reducing fat accumulation in the liver and slowing NAFLD progression. First, elevated basal metabolic rate due to increased skeletal muscle mass leads to higher caloric consumption, which is involved in weight loss and fatty liver improvement^29^. Second, skeletal muscle is one of the key players in glucose metabolism. The skeletal muscle is a target organ of insulin, and increased muscle mass may be related to improved glucose metabolism capacity. In addition, a study on a skeletal muscle-specific, conditional transgenic mice reported that increased muscle mass prevented insulin resistance, which is one of the key mechanisms of NAFLD^30^.

Myokines from skeletal muscle also play important roles in the sarcopenia-NAFLD relationship. They control the hepatic metabolism of glucose and fatty acids and have anti-inflammatory effects against the proinflammatory activation of adipocytes^31,32^. The association between the change in skeletal muscle mass and NAFLD progression can also be explained in terms of malnutrition, which is a known cause of NAFLD^33^. Since sarcopenia is often accompanied by malnutrition, increased skeletal muscle mass in these patients may suggest an improvement in nutritional status and accompanying resolution of the cause of NAFLD exacerbation^34^. Considering the above-mentioned, treatment for sarcopenia may be a potential therapeutic intervention for NAFLD^35^. Therefore, investigating the effect of sarcopenia-targeted therapy on NAFLD status through subsequent research may be helpful in expanding NAFLD treatment strategies in the future.

In the absence of approved pharmacological treatment, lifestyle modification including exercise is central to the treatment of NAFLD. Exercise can reduce intrahepatic fat content and serum aminotransferase level even without significant weight loss^36,37^. Exercise showed a beneficial effect on functional capacity in patients with NAFLD^38^. Additionally, exercise also reduces muscle loss and prevents progression of sarcopenia^39,40^. Therefore, exercise can play an important role as a therapeutic intervention in patients with NAFLD and patients with sarcopenia. However, more studies are needed to determine the optimal exercise type, intensity, and duration to improve sarcopenia and NAFLD.

Consensus guidelines on sarcopenia suggested various methods to assess the skeletal muscle mass^41,42^. While magnetic resonance imaging, computed tomography scans, and dual-energy x-ray absorptiometry (DEXA) are regarded as reliable reference techniques for the ASM measurement, their accessibility in clinical settings is limited, and both CT and DXA can entail exposure to radiation^43^. Alternatively, the impedance measurement has advantages in terms of being relatively inexpensive, rapid, and portable without carrying the risk of radiation exposure. In several studies comparing the accuracy of lean soft tissue mass measurement with DEXA, impedance measurements showed satisfactory performance against DEXA^43,44^. Considering the appropriate diagnostic performance and good accessibility, impedance measurements are expected to play a crucial role in the clinical field of sarcopenia.

In studies on sarcopenia, height, body weight, and BMI have been mainly used to adjust ASM measurements. According to the AWGS2019 guideline, BMI-adjusted muscle mass was recommended for the evaluation of sarcopenia in clinical and research fields^42^, but there is still a lack of studies that compare weight-adjusted ASM and BMI-adjusted ASM with long-term data. In a study that investigated sarcopenia and hepatic fibrosis, there was no apparent difference between ASM/Weight and ASM/BMI. This was consistent with our results^16^. At present, we suggest that both ASM/Weight and ASM/BMI can be applicable in clinical and research settings for sarcopenia in patients with NAFLD.

As muscle mass typically declines with aging, it is expected that the proportion of patients with sarcopenia in the same group will increase over time. In our data, patterns of increasing sarcopenia rates were confirmed with a time interval of 6 years as expected. However, the data did not show statistical significance (Table 2). Considering that relatively healthy adults were set as the study population, we also concluded that the effect of aging alone on the sarcopenic state of the study group was insignificant.

Hepatic fibrosis is one of the most important factors determining the long-term prognosis of NAFLD. This study aimed to determine the potential impact of sarcopenia on the progression of hepatic fibrosis. Although the increase in NFS over 6 years was higher in patients with baseline sarcopenia, no significant association between sarcopenia and changes in FIB-4 was shown in our analyses. Since a patient’s age is included in the formula for NFS and FIB4, these two indices tend to increase over time as the patient ages^24^. However, contrary to our expectations, the values of the study population were lower after 6 years, and the correlation with the change in skeletal muscle mass was not significant, even after adjusting for age, sex, and metabolic syndrome.

These results can be attributed to several factors. First, the potential factors related to hepatic fibrosis may not have been sufficiently controlled in this study. Since patients who visited at least twice for health screening were targeted, the health-related interests and efforts of the study subjects may be higher than those of the general population. Therefore, the variables included in the calculation of fibrosis indices, other than age, may be improved over 6 years, making NFS and FIB4 better than predicted. Another possible reason for our unexpected results is that the 6-year time interval between the two observation points may not be sufficient for observing significant changes in hepatic fibrosis. According to a meta-analysis study targeting liver biopsy-based studies, the time required for the progression from baseline stage 0 fibrosis to hepatic fibrosis stage 1 was approximately 14.3 years in NAFLD patients, and 7.1 years in patients with NASH^45^. Since this study was conducted on asymptomatic patients visiting for health screening, the majority of the study group may have NAFLD rather than NASH. Therefore, it may take more than 6 years to identify the progression of fibrosis.

This study had several limitations. First, for the evaluation of hepatic steatosis, HSI and FLI—which are noninvasive serum markers—were chosen. Although these markers are widely used in clinical fields due to their convenience, with validated diagnostic performances in previous studies, their limitations are that they are less accurate and unable to quantitatively evaluate hepatic histologic changes, compared to US or magnetic resonance imaging^46,47^. Additionally, NFS and FIB4, which represent hepatic fibrosis, are known to have a lower accuracy than biopsy- or imaging-based methods^48,49^. In future studies investigating the impact of sarcopenia on liver diseases, we recommend a test method that can more directly evaluate hepatic steatosis or fibrosis.

Second, since we only collected data at two time points (with a 6-year interval), continuous follow-up for skeletal muscle mass and each liver-related index was not possible. Therefore, the identification of the changes between the two time points is limited. Third, lifestyle-related factors such as alcohol consumption, cigarette smoking, or exercise may have influenced the sarcopenia status of the study population. Regarding alcohol consumption, we defined the study population as patients with non-alcoholic fatty liver disease. As patients with alcohol consumption above a certain level were excluded, the possibility that the drinking habit had an effect on the sarcopenia status of the study group may be minimal. Unfortunately, due to the nature of the retrospective study, information on smoking history and exercise-related factors could not be obtained. In future related studies, it will be necessary to investigate and analyze detailed lifestyle variables.

Fourth, although the patient’s history of diabetes was included as a variable in our study, we were unable to suggest a parameter that could quantify insulin resistance, such as the Homeostatic Model Assessment for Insulin Resistance (HOMA-IR). Considering well-known associations between insulin resistance & NAFLD and sarcopenia, objective indicators reflecting insulin resistance may be required for future studies of NAFLD. Measurement of serum insulin levels for NAFLD patients as a daily practice may also be considered noteworthy.

Lastly, since the patients included were relatively healthy, there is an overall lack of patients with advanced fibrosis, to show a significant conclusion in temporal change of hepatic fibrosis, even in non-invasive setting. Furthermore, insufficient sample size can be one of the reasons for inconsistent relationships between hepatic fibrosis and sarcopenia in our data; therefore, future researches including patients with more advanced fibrosis will be needed.

In conclusion, skeletal muscle loss can accelerate the exacerbation of hepatic steatosis in patients with NAFLD, and vice versa. It is worth noting that improvements in sarcopenia may be a therapeutic target in the treatment of NAFLD. In future studies, it may be necessary to concentrate on the effects of therapeutic interventions for sarcopenia on NAFLD status, with a prospective design.

## Supplementary Information

Supplementary Tables.

## Data Availability

The data that support the findings of this study are available on request from the corresponding author.
