# Supplementary material for: Change in skeletal muscle mass is associated with hepatic steatosis in nonalcoholic fatty liver disease
Source: Sci Rep. 2023 Apr 28;13:6920. doi: 10.1038/s41598-023-34263-z (PMC10147659; doi:10.1038/s41598-023-34263-z)
Supplement: Supplementary file 1 — Supplementary Tables. [file 41598_2023_34263_MOESM1_ESM.docx]

Supplement Table S1. Subgroup Differences between the two groups according to the muscle mass reduction after 6 years from baseline visit (subgroup analysis for those with sarcopenia by ASM/BMI at baseline visit, **Bold: p value < 0.05**).

|  | Reduced ASM/BMI (-) N=20 | Reduced ASM/BMI (+) N=22 | p |
| --- | --- | --- | --- |
| Age (years) | 51.4 ± 8.6 | 52.9 ± 12.4 | 0.650 |
| Gender: Male | 15 (75.0%) | 18 (81.8%) | 0.872 |
| BMI (kg/m^2^) | 26.7 ± 2.4 | 28.3 ± 3.2 | 0.080 |
| Diabetes | 3 (15.0%) | 2 (9.1%) | 0.910 |
| Hypertension | 9 (45.0%) | 12 (54.5%) | 0.757 |
| Metabolic syndrome | 15 (75.0%) | 13 (59.1%) | 0.444 |
| Smoking: Yes | 5 (25.0%) | 6 (27.3%) | 1.000 |
| Alcohol use: Yes | 8 (40.0%) | 11 (50.0%) | 0.734 |
| Changes for 6 years in liver-related index |  |  |  |
| **Δ**NFS | 0.8 ± 0.8 | 0.9 ± 0.7 | 0.731 |
| **Δ**FIB4 | 0.3 ± 0.3 | 0.3 ± 0.3 | 0.526 |
| **Δ**HSI | -1.5 ± 2.7 | -1.3 ± 4.6 | 0.813 |
| **Δ**FLI | -6.2 ± 22.0 | 3.2 ± 22.2 | 0.178 |
| Changes for 6 years in Laboratory findings |  |  |  |
| ΔWBC (10^9^/L) | -0.3 ± 1.6 | -0.1 ± 1.8 | 0.766 |
| ΔHemoglobin (g/dL) | -0.2 ± 1.6 | 0.1 ± 1.0 | 0.475 |
| **Δ**Platelet (10^9^/L) | -9.8 ± 33.8 | -1.4 ± 22.4 | 0.342 |
| ΔGlucose (mg/dL) | 7.2 ± 15.1 | 14.0 ± 21.3 | 0.244 |
| ΔProtein (g/dL) | -0.2 ± 0.5 | 0.1 ± 0.3 | 0.055 |
| ΔAlbumin (g/dL) | -0.1 ± 0.4 | -0.0 ± 0.3 | 0.499 |
| **Δ**BUN (mg/dL) | -2.1 ± 3.8 | -0.7 ± 4.5 | 0.287 |
| **Δ**Creatinine (mg/dL) | -0.1 ± 0.2 | -0.1 ± 0.1 | 0.961 |
| **Δ**Sodium (mEq/L) | -8.1 ± 31.6 | 5.1 ± 30.7 | 0.178 |
| **Δ**Potassium (mEq/L) | -0.2 ± 0.9 | 0.2 ± 0.9 | 0.184 |
| **Δ**Total bilirubin (mg/dL) | 0.1 ± 0.4 | 0.1 ± 0.3 | 0.475 |
| ΔAST (IU/L) | 3.0 ± 11.7 | 2.9 ± 11.7 | 0.980 |
| ΔALT (IU/L) | -2.9 ± 12.2 | -4.7 ± 23.8 | 0.759 |
| **Δ**ALP (IU/L) | -129.3 ± 40.0 | -130.0 ± 46.0 | 0.959 |
| ΔGGT (U/L) | -2.2 ± 12.9 | -6.1 ± 30.3 | 0.587 |
| **Δ**Uric acid (mg/dL) | -0.0 ± 1.2 | -0.3 ± 0.8 | 0.437 |
| ΔTotal cholesterol (mg/dL) | -46.8 ± 55.5 | -22.9 ± 52.6 | 0.161 |
| ΔTriglyceride (mg/dL) | -102.8 ± 232.8 | -22.8 ± 79.6 | 0.158 |
| **Δ**HDL cholesterol (mg/dL) | 3.5 ± 9.6 | 2.0 ± 9.0 | 0.593 |
| ΔLDL cholesterol (mg/dL) | -19.1 ± 47.1 | -4.1 ± 49.6 | 0.323 |
| ASM: Appendicular skeletal muscle mass, BMI: Body mass index, NFS: NAFLD Fibrosis Score, FIB4: Fibrosis-4 Index for Liver Fibrosis, HSI: Hepatic Steatosis Index, FLI: Fatty Liver Index, WBC: White blood cell, BUN: Blood urea nitrogen, AST: Aspartate aminotransferase, ALT: Alanine aminotransferase, ALP: Alkaline phosphatase, GGT: Gamma-glutamyl transferase, HDL: High-density lipoprotein, LDL: Low-density lipoprotein | | | |

Supplement Table S2. Changes in liver-related indices for 6 years according to the baseline sarcopenia status and gender **(Bold: p value < 0.05)**

|  |  | ASM/Weight | |  | ASM/BMI | |  |
| --- | --- | --- | --- | --- | --- | --- | --- |
|  |  | Sarcopenia (+) | Sarcopenia (-) | p | Sarcopenia (+) | Sarcopenia (-) | p |
| Male | ΔNFS | **0.9 ± 0.6** | **0.5 ± 0.8** | **0.002** | **1.0 ± 0.7** | **0.5 ± 0.8** | **0.001** |
|  | ΔFIB4 | 0.3 ± 0.2 | 0.3 ± 0.3 | 0.946 | 0.3 ± 0.3 | 0.3 ± 0.3 | 0.847 |
|  | ΔHSI | -1.7 ± 3.4 | -1.0 ± 3.6 | 0.243 | -1.9 ± 3.9 | -1.0 ± 3.5 | 0.156 |
|  | ΔFLI | -6.6 ± 16.1 | -0.1 ± 17.0 | 0.024 | -6.0 ± 15.6 | -0.3 ± 17.1 | 0.063 |
| Female | ΔNFS | 0.5 ± 0.9 | 0.3 ± 0.8 | 0.320 | 0.2 ± 0.9 | 0.3 ± 0.8 | 0.772 |
|  | ΔFIB4 | 0.2 ± 0.2 | 0.3 ± 0.3 | 0.910 | 0.2 ± 0.3 | 0.3 ± 0.3 | 0.958 |
|  | ΔHSI | -0.0 ± 3.9 | -0.2 ± 3.3 | 0.826 | 0.6 ± 2.2 | -0.2 ± 3.4 | 0.466 |
|  | ΔFLI | 9.4 ± 32.7 | 5.1 ± 16.0 | 0.622 | 16.0 ± 34.0 | 4.9 ± 16.5 | 0.360 |

ASM: Appendicular skeletal muscle mass, BMI: Body mass index, NFS: NAFLD Fibrosis Score, FIB4: Fibrosis-4 Index for Liver Fibrosis, HIS: Hepatic Steatosis Index, FLI: Fatty Liver Index

Supplement Table S3 Changes in liver-related indices for 6 years according to the baseline sarcopenia status and age **(Bold: p value < 0.05)**

|  |  | ASM/Weight | |  | ASM/BMI | |  |
| --- | --- | --- | --- | --- | --- | --- | --- |
|  |  | Sarcopenia (+) | Sarcopenia (-) | p | Sarcopenia (+) | Sarcopenia (-) | p |
| Age 50 or over | ΔNFS | **0.9 ± 0.5** | **0.6 ± 0.7** | **0.008** | **0.9 ± 0.6** | **0.5 ± 0.7** | **0.030** |
|  | ΔFIB4 | 0.4 ± 0.3 | 0.4 ± 0.4 | 0.839 | 0.3 ± 0.2 | 0.4 ± 0.4 | 0.427 |
|  | ΔHSI | -0.8 ± 3.7 | -0.9 ± 2.9 | 0.870 | -1.6 ± 4.0 | -0.8 ± 2.9 | 0.382 |
|  | ΔFLI | -2.3 ± 16.8 | -1.1 ± 16.1 | 0.729 | -5.1 ± 16.5 | -0.8 ± 16.1 | 0.216 |
| Age under 50 | ΔNFS | **0.8 ± 0.9** | **0.4 ± 0.8** | **0.015** | 0.8 ± 1.0 | 0.4 ± 0.8 | 0.070 |
|  | ΔFIB4 | 0.2 ± 0.2 | 0.2 ± 0.3 | 0.704 | 0.3 ± 0.4 | 0.2 ± 0.3 | 0.835 |
|  | **Δ**HSI | -1.6 ± 3.5 | -0.7 ± 3.8 | 0.201 | -1.2 ± 3.5 | -0.7 ± 3.8 | 0.628 |
|  | **Δ**FLI | -2.0 ± 26.6 | 3.1 ± 17.1 | 0.302 | 3.7 ± 28.1 | 2.6 ± 17.5 | 0.871 |

ASM: Appendicular skeletal muscle mass, BMI: Body mass index, NFS: NAFLD Fibrosis Score, FIB4: Fibrosis-4 Index for Liver Fibrosis, HIS: Hepatic Steatosis Index, FLI: Fatty Liver Index

Supplement Table S4. Regression analysis of muscle mass variances as predictors for changes in liver-related indices over 6 years, stratified by gender, adjusted by age, diabetes, hypertension, and metabolic syndrome **(Bold: p value < 0.05)**

|  |  | **Δ**ASM/Weight | | | | | **Δ**ASM/BMI | | | | |  |
| --- | --- | --- | --- | --- | --- | --- | --- | --- | --- | --- | --- | --- |
|  |  |  | 95% CI | |  | | |  | 95% CI | |  |  |
|  |  | Estimate | Lower Limit | Upper Limit | | p | | Estimate | Lower Limit | Upper Limit | p | |
| **Male** | **Δ**NFS | 0.032 | -0.019 | 0.083 | | 0.223 | | 0.817 | -0.802 | 2.434 | 0.322 | |
|  | **Δ**FIB4 | 0.012 | -0.010 | 0.033 | | 0.278 | | 0.410 | -0.266 | 1.087 | 0.234 | |
|  | **Δ**HSI | **-0.908** | -1.132 | -0.684 | | **<0.001** | | **-26.567** | -33.731 | -19.403 | **<0.001** | |
|  | **Δ**FLI | **-5.462** | -6.425 | -4.500 | | **<0.001** | | **-160.740** | -191.832 | -129.649 | **<0.001** | |
| **Female** | **Δ**NFS | -0.006 | -0.094 | 0.082 | | 0.896 | | -0.351 | -3.574 | 2.872 | 0.830 | |
|  | **Δ**FIB4 | 0.024 | -0.004 | 0.052 | | 0.093 | | 0.794 | -0.229 | 1.817 | 0.128 | |
|  | **Δ**HSI | **-0.994** | -1.317 | -0.674 | | **<0.001** | | **-34.187** | -46.162 | -22.213 | **<0.001** | |
|  | **Δ**FLI | **-4.550** | -6.300 | -2.800 | | **<0.001** | | **-161.032** | -225.463 | -96.601 | **<0.001** | |

CI: Confidential interval, ASM: Appendicular skeletal muscle mass, BMI: Body mass index, NFS: NAFLD Fibrosis Score, FIB4: Fibrosis-4 Index for Liver Fibrosis, HIS: Hepatic Steatosis Index, FLI: Fatty Liver Index

Supplement Table S5. Regression analysis of muscle mass variances as predictors for changes in liver-related indices over 6 years, stratified by age (under 50 or not), adjusted by gender, diabetes, hypertension, and metabolic syndrome **(Bold: p value < 0.05)**

|  |  | **Δ**ASM/Weight | | | | **Δ**ASM/BMI | | | | | |  |
| --- | --- | --- | --- | --- | --- | --- | --- | --- | --- | --- | --- | --- |
|  |  | 95% CI | | | | | 95% CI | | | | |  |
|  |  | Estimate | Lower Limit | Upper Limit | p | | | Estimate | Lower Limit | Upper Limit | p | |
| **Age 50 or over** | **Δ**NFS | 0.043 | -0.022 | 0.108 | 0.195 | | | 1.067 | -1.135 | 3.268 | 0.341 | |
|  | **Δ**FIB4 | 0.009 | -0.023 | 0.041 | 0.569 | | | 0.111 | -0.974 | 1.196 | 0.840 | |
|  | **Δ**HSI | **-0.498** | -0.756 | -0.242 | **<0.001** | | | **-16.047** | -24.741 | -7.353 | **<0.001** | |
|  | **Δ**FLI | **-3.941** | -5.276 | -2.607 | **<0.001** | | | **-133.883** | -178.788 | -88.979 | **<0.001** | |
| **Age under 50** | **Δ**NFS | -0.005 | -0.066 | 0.056 | 0.872 | | | -0.167 | -2.147 | 1.813 | 0.869 | |
|  | **Δ**FIB4 | 0.0165 | -0.003 | 0.036 | 0.097 | | | **0.660** | 0.032 | 1.289 | **0.040** | |
|  | **Δ**HSI | **-1.236** | -1.488 | -0.985 | **<0.001** | | | **-36.065** | -44.389 | -27.742 | **<0.001** | |
|  | **Δ**FLI | **-6.1737** | -7.319 | -5.028 | **<0.001** | | | **-179.253** | -217.323 | -141.184 | **<0.001** | |

CI: Confidential interval, ASM: Appendicular skeletal muscle mass, BMI: Body mass index, NFS: NAFLD Fibrosis Score, FIB4: Fibrosis-4 Index for Liver Fibrosis, HIS: Hepatic Steatosis Index, FLI: Fatty Liver Index

Supplement Table S6. Regression analysis of muscle mass variances as predictors for changes in liver-related indices over 6 years, stratified by the presence of diabetes, adjusted by gender, hypertension, and metabolic syndrome **(Bold: p value < 0.05)**

|  |  | **Δ**ASM/Weight | | | | **Δ**ASM/BMI | | | | | |  |
| --- | --- | --- | --- | --- | --- | --- | --- | --- | --- | --- | --- | --- |
|  |  | 95% CI | | | | | 95% CI | | | | |  |
|  |  | Estimate | Lower Limit | Upper Limit | p | | | Estimate | Lower Limit | Upper Limit | p | |
| **With diabetes** | **Δ**NFS | 0.049 | -0.154 | 0.252 | 0.633 | | | 1.719 | -4.570 | 8.008 | 0.587 | |
|  | **Δ**FIB4 | -0.033 | -0.115 | 0.049 | 0.427 | | | -0.854 | -3.406 | 1.696 | 0.505 | |
|  | **Δ**HSI | -0.278 | -1.074 | 0.519 | 0.489 | | | -0.323 | -25.111 | 24.465 | 0.979 | |
|  | **Δ**FLI | **-4.420** | -7.976 | -0.863 | **0.016** | | | **-114.901** | -226.709 | -3.093 | **0.044** | |
| **Without diabetes** | **Δ**NFS | 0.015 | -0.031 | 0.060 | 0.532 | | | 0.275 | -1.235 | 1.785 | 0.720 | |
|  | **Δ**FIB4 | **0.018** | 0.001 | 0.035 | **0.042** | | | **0.582** | 0.010 | 1.155 | **0.046** | |
|  | **Δ**HSI | **-0.951** | -1.141 | -0.762 | **<0.001** | | | **-29.473** | -35.795 | -23.152 | **<0.001** | |
|  | **Δ**FLI | **-5.229** | -6.120 | -4.337 | **<0.001** | | | **-162.364** | -192.208 | -132.519 | **<0.001** | |

CI: Confidential interval, ASM: Appendicular skeletal muscle mass, BMI: Body mass index, NFS: NAFLD Fibrosis Score, FIB4: Fibrosis-4 Index for Liver Fibrosis, HIS: Hepatic Steatosis Index, FLI: Fatty Liver Index
